# IGF-1 Controls Metabolic Homeostasis and Survival in HEI-OC1 Auditory Cells through AKT and mTOR Signaling

**DOI:** 10.3390/antiox12020233

**Published:** 2023-01-19

**Authors:** Ángela García-Mato, Blanca Cervantes, Lourdes Rodríguez-de la Rosa, Isabel Varela-Nieto

**Affiliations:** 1 Neuropathology of Hearing and Myelinopathies Group, Institute for Biomedical Research “Alberto Sols”, Spanish National Research Council-Autonomous University of Madrid (CSIC-UAM), 28029 Madrid, Spain; 2 Consorcio Centro de Investigación Biomédica en Red (CIBERER), Institute of Health Carlos III (ISCIII), 28029 Madrid, Spain; 3 School of Medicine, University Anáhuac Puebla, Puebla 72810, Mexico; 4 Hospital La Paz Institute for Health Research (IdiPAZ), 28029 Madrid, Spain

**Keywords:** anabolism, apoptosis, autophagy, cisplatin, IGF1R, NRF2, otic differentiation, oxidative stress

## Abstract

Insulin-like growth factor 1 (IGF-1) is a trophic factor for the nervous system where it exerts pleiotropic effects, including the regulation of metabolic homeostasis. IGF-1 deficiency induces morphological alterations in the cochlea, apoptosis and hearing loss. While multiple studies have addressed the role of IGF-1 in hearing protection, its potential function in the modulation of otic metabolism remains unclear. Here, we report that “House Ear Institute-organ of Corti 1” (HEI-OC1) auditory cells express IGF-system genes that are regulated during their differentiation. Upon binding to its high-affinity receptor IGF1R, IGF-1 activates AKT and mTOR signaling to stimulate anabolism and, concomitantly, to reduce autophagic catabolism in HEI-OC1 progenitor cells. Notably, IGF-1 stimulation during HEI-OC1 differentiation to mature otic cells sustained both constructive metabolism and autophagic flux, possibly to favor cell remodeling. IGF1R engagement and downstream AKT signaling promoted HEI-OC1 cell survival by maintaining redox balance, even when cells were challenged with the ototoxic agent cisplatin. Our findings establish that IGF-1 not only serves an important function in otic metabolic homeostasis but also activates antioxidant defense mechanisms to promote hair cell survival during the stress response to insults.

## 1. Introduction

Insulin-like growth factor 1 (IGF-1) is a trophic factor that has a crucial role in the regulation of developmental cell growth and differentiation and in metabolism in the central nervous system. It is essential for the formation of circuits involved in the control of brain energy homeostasis [1] and, in combination with insulin, for glucose handling by astrocytes [2]. IGF-1 is also crucial for the differentiation of neural stem cells and progenitors to mature neurons during hippocampal neurogenesis [3], for photoreceptor neuroprotection by maintaining the structure, functionality and energy homeostasis of photoreceptors [4], and for counteracting the effects of retinal inflammation [5]. Loss-of-function mutations in the gene coding for IGF-1 cause an ultrarare human hearing loss syndrome [6,7]. Similarly, mice deficient for *Igf1* show hearing loss [8,9,10], whereas haploinsufficiency causes chronic inflammation and oxidative stress and accelerates age-related hearing loss [11,12]. 

IGF-1 belongs to the IGF system, together with other ligands (insulin and IGF-2), transmembrane receptors (insulin receptor (IR) and insulin-like growth factor receptors type 1 (IGF1R) and type 2 (IGF2R)) and IGF-binding proteins (IGFBP1–6) [7,13]. The expression of IGF-1, IGF-2, IGF1R and IGFBP2 has been reported in the developing [14,15,16] and postnatal [8,12,17,18,19] cochlea with cell-type-specific spatiotemporal patterns.

**Figure 1:**
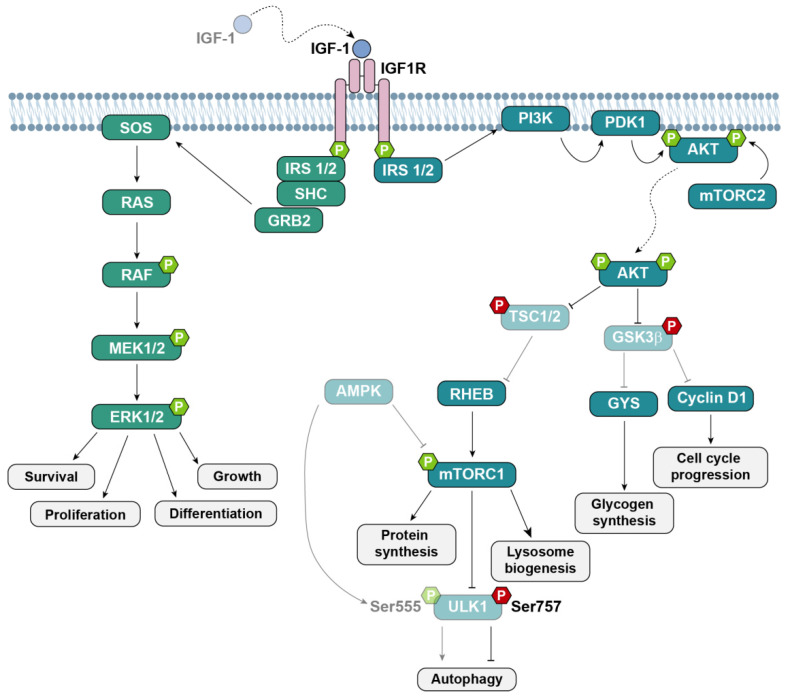
IGF-1 signaling controls several pathways through the IGF1R/AKT and IGF1R/ERK cascades. Binding of IGF-1 to IGF1R leads to receptor activation through autophosphorylation. Activated IGF1R, in turn, phosphorylates several docking proteins, which serve as signaling nodes for the activation of two main pathways: the PI3K/AKT/mTOR pathway and the ERK MAPK cascade. Both pathways are involved in the regulation of highly relevant cellular processes such as the proliferation, survival and synthesis of macromolecules. P indicates phosphorylation sites, and red and green hexagons indicate inhibition and activation, respectively, by phosphorylation.

Upon the binding of IGF-1, the high-affinity IGF1R is phosphorylated and recruits and phosphorylates adaptor proteins that, in turn, facilitate the activation of a network of downstream signaling molecules to promote the activation of the extracellular signal-regulated kinase (ERK), mitogen-activated protein kinase (MAPK) and phosphatidylinositol 3-kinase (PI3K)–thymoma viral proto-oncogene (AKT) pathways [20] (Figure 1).

The activation of AKT signaling is essential for auditory hair cell survival in response to ototoxic insults [21,22,23,24,25] and during aging [26]. Furthermore, IGF-1, via IGF1R and AKT/ C-RAF/ERK activation, modulates early chicken inner ear neurogenesis and differentiation [27,28,29,30], and this activity is essential for the onset and conservation of mammalian hearing [10,14,31,32]. 

AKT is, *per se*, a signaling node that regulates key cellular processes, including macromolecule synthesis and cell survival, by modulating multiple targets through its serine/threonine kinase activity. Once activated by dual phosphorylation, AKT can inhibit the constitutive activity of glycogen synthase kinase-3β (GSK3β) by phosphorylating serine (Ser)9, consequently promoting glycogen synthesis and storage and stimulating G1/S cell cycle transition [20,33]. AKT also modulates the activation of the mammalian target of rapamycin (mTOR), which forms part of mTOR complex 1 (mTORC1), a serine/threonine kinase that regulates cell metabolism by favoring protein, lipid and nucleotide synthesis and inhibiting autophagy [33,34]. Activated mTOR phosphorylates and activates the ribosomal protein S6 kinase B1 (p70S6K) to promote protein synthesis [34]. Beyond AKT activation, mTORC1 activity can be modulated by the physiological energy sensor adenosine monophosphate (AMP)-activated protein kinase (AMPK). A shared downstream target of AMPK and mTOR is the uncoordinated protein (Unc)-51-like autophagy activating kinase (ULK1), which is required for autophagy initiation (Figure 1). Upon stimulation by growth factors, AKT activates mTOR, which, in turn, inhibits ULK1 by phosphorylation on Ser757, thus blocking autophagy initiation. By contrast, in an energy-depleted scenario, the increase in the AMP/ATP ratio promotes AMPK activation, which suppresses anabolism through the inhibition of mTOR and redirects metabolism towards catabolic processes such as autophagy by phosphorylating ULK1 on Ser555 [35]. Under stress conditions, energy homeostasis controlled by mTORC1 and AMPK is crucial for sensory hair cell survival [36,37].

The organ of Corti is the hearing receptor of the cochlea and contains a limited number of post-mitotic sensory hair cells. The chemotherapeutic agent cisplatin (*cis*-diammine-dichloroplatinum (II)) is widely used in the treatment of solid tumors but has significant ototoxic side effects, including hair cell oxidative stress, DNA damage and apoptosis [38,39]. Cisplatin-induced hearing loss is bilateral, progressive and irreversible, which hinders its therapeutic anti-cancer profile [40]. IGF-1 has been shown to be otoprotective against multiple ototoxic insults [22,23,41,42], but whether it is otoprotective against cisplatin is not known.

IGF-1 is essential for brain metabolism and normal hearing; however, no studies have addressed its contribution to cochlear metabolic homeostasis. Here, we show that IGF-1 signaling in progenitor HEI-OC1 auditory cells promotes survival and anabolism, maintaining redox balance and cell proliferation while reducing autophagic flux and apoptosis. We found that IGF-1 supports survival and promotes anabolism during the early differentiation of HEI-OC1 cells to mature otic cells, but it does not regulate autophagic flux, so cell remodeling is facilitated. IGF-1 also protects HEI-OC1 cells against ototoxic insults using similar downstream mechanisms mediated by AKT activation, supporting the antioxidant cellular response.

## 2. Materials and Methods

### 2.1. Cell Culture and Treatments

The House Ear Institute-organ of Corti 1 (HEI-OC1) auditory cell line was kindly provided by Dr. Federico Kalinec (UCLA, Department of Head and Neck Surgery, Los Angeles, CA, USA). Cells were cultured under permissive (at 33 °C and 10% CO_2_) or non-permissive (at 39 °C and 5% CO_2_) conditions in high-glucose Dulbecco’s modified Eagle’s medium (DMEM, Gibco, Thermo Fisher Scientific, Waltham, MA, USA) containing 10% fetal bovine serum (FBS, Gibco) without antibiotics, as previously described [43]. HEI-OC1 cells maintained under permissive conditions proliferate and are considered progenitor cells, whereas cells cultured under non-permissive conditions become post-mitotic and rapidly differentiate into hair-cell-like organ of Corti cells, which is irreversible [43]. Because differentiation also triggers apoptosis, most studies have focused on progenitor HEI-OC1 cells and not on differentiated HEI-OC1 cells [43,44].

Cells were treated with the following agents: (i) human recombinant IGF-1 (PeproTech, Thermo Fisher Scientific); (ii) bafilomycin A1 (BAF-A1; Sigma-Aldrich, Saint Louis, MO, USA); (iii) IGF1R inhibitor NVP-AEW541 (NVP; Cayman Chemical, Ann Arbor, MI, USA); (iv) cisplatin (Accord Healthcare Ltd., Middlesex, UK); or (v) their combinations, as indicated. DMSO was used as a solvent and had no detectable effects at the doses used (up to 0.07%). To enable cell cycle synchronization [45] and to test the effect of exogenous IGF-1, cells were cultured in serum-free DMEM when indicated. Because hair cells are post-mitotic, in order to study cisplatin toxicity and IGF-1 otoprotection, we used serum deprivation to induce quiescence in progenitors [45,46].

### 2.2. Immunofluorescence

Cells were plated onto glass coverslips and cultured for 4 days. Cells were then fixed with 2% paraformaldehyde (Merck, Kenilworth, NJ, USA), washed with 0.1 M phosphate-buffered saline (PBS) pH 7.4 and permeabilized and blocked with 0.1% Triton X-100 (Sigma-Aldrich), 5% normal goat serum or donkey serum (Sigma-Aldrich) and 0.2% bovine serum albumin (NZYTech, Lisbon, Portugal) in PBS (PBS-T). Cells were then incubated overnight at 4 °C with primary antibodies (Table S1) diluted in PBS-T. Subsequently, cells were washed and incubated with an Alexa Fluor^®^-conjugated secondary antibody (1:500), Alexa Fluor^®^ 546 phalloidin (1:250) and DAPI (1:1000) (all from Thermo Fisher Scientific) diluted in PBS-T for 2 h at room temperature (RT). Preparations were mounted in a ProLong^®^ Diamond device (Thermo Fisher Scientific) and visualized using a Zeiss LSM710 confocal microscope (Carl Zeiss, Oberkochen, Germany), and images were processed with Fiji software (National Institutes of Health, Bethesda, MD, USA).

### 2.3. RNA Isolation and RT-qPCR

RNA extraction was performed using the NZY Total RNA Isolation Kit (NZYTech), and RNA integrity was assessed with an Agilent 2100 bioanalyzer (Agilent Technologies, Santa Clara, CA, USA). cDNA was generated from RNA by reverse transcription with the High-Capacity cDNA Reverse Transcription Kit (Applied Biosynthesis, Thermo Fisher Scientific) and amplified by quantitative (q) PCR in a 7900 HT FAST real-time PCR system (Applied Biosynthesis) or in a QS7 Flex real-time PCR system (Applied Biosynthesis) using either commercial TaqMan^®^ probes or gene-specific primers (Tables S2 and S3). *Hprt1* (hypoxanthine phosphoribosyltransferase 1) and *Rplp0* (ribosomal protein lateral stalk subunit P0) genes were used as housekeeping genes for normalization, and the estimated gene expression was calculated as 2^−ΔCt^ or 2^−ΔΔCt^ when indicated, as reported [47].

### 2.4. Protein Extraction and Western Blotting

Western blotting was performed using protein extracts from cells lysed in a buffer containing 1.5 mM MgCl_2_, 0.2 mM EDTA, 0.3 M NaCl, 25 mM HEPES pH 7.5, 0.1% Triton X-100 (Sigma-Aldrich), a phosphatase and protease inhibitor cocktail (Sigma-Aldrich) and 1 mM DTT (Roche Molecular Systems, Pleasanton, CA, USA). Protein concentration was quantified using the Bradford Assay (Bio-Rad Laboratories, Hercules, CA, USA). Equal amounts of protein were subjected to gel electrophoresis on 8, 10, 12 or 14% SDS-PAGE gels and were transferred to PVDF membranes (0.2 µm) using a Bio-Rad Trans Blot TURBO instrument (Bio-Rad Laboratories). After incubation with 5% bovine serum albumin or non-fat milk, membranes were probed overnight at 4 °C with the primary antibodies and then with the corresponding peroxidase-conjugated secondary antibody for 1 h at RT (Table S1). The PI3K p85 regulatory subunit was used as a loading control as it shows stable expression in multiple cell types, even in response to IGF-1 [48]. Immunoreactive bands were visualized using the Clarity™ Western ECL Substrate (Bio-Rad Laboratories), and images were captured and quantified on an ImageQuant LAS4000 analyzer (GE Healthcare, Fairfield, CT, USA). Different exposure times were used to ensure that the bands were not saturated.

### 2.5. Cell Viability 

Cells were seeded on 96-well flat-bottom plates and incubated for 24 h. Cell viability was determined with the Cell Proliferation Kit II (XTT) (Roche Molecular Systems), as reported [39,49]. Optical density was measured with a VERSAmax^TM^ tunable microplate reader running SOFTmax^®^ Pro 3.0 software (Molecular Devices, Sunnyvale, CA, USA), and average optical density in the control experimental group was taken as 100% of cell viability. In experiments investigating the effects of cisplatin, the cell viability in the control condition (no cisplatin) was used to correct for the base level of the apoptosis induced by serum deprivation.

### 2.6. TUNEL Assay

Apoptosis was evaluated using the Dead-End^TM^ Fluorometric TUNEL System (Promega, Madison, WI, USA). Cells were fixed with 2% paraformaldehyde and permeabilized with 66% ice-cold ethanol for 2 h at 4 °C. Subsequently, the cells were rinsed with PBS and incubated in equilibration buffer for 5 min at RT. Fixed cells were incubated with fluorescein-12-dUTP in a reaction catalyzed by recombinant terminal deoxynucleotidyl transferase (TdT) for 1 h at 37 °C. After washing, cells were incubated with propidium iodide (PI)/RNase Staining Solution (Cell Signaling Technology, Danvers, MA, USA) for 30 min at RT in darkness. Stained cells were analyzed by flow cytometry using a Cytomics FC 500 MPL system and quantified with MXP software (both from Beckman Coulter Brea, CA, USA).

### 2.7. Annexin V-FITC and Propidium Iodine Dual Staining

Annexin V-FITC (Immunostep, Salamanca, Spain) and PI (Abcam, Cambridge, UK) double labeling was used to quantify the number of apoptotic cells. Cells were washed with PBS and suspended in binding buffer (10 mM HEPES/NaOH, pH 7.4), 140 mM NaCl and 2.5 mM CaCl_2_ (Immunostep). Next, cells were labeled with annexin V-FITC/PI for 15 min at RT in darkness and then analyzed by flow cytometry using a Cytomics FC 500 MPL system (Beckman Coulter), as reported [39,49].

### 2.8. DNA Oxidative Damage Detection

Levels of DNA oxidative damage were measured with the EpiQuik^TM^ 8-OhdG DNA Damage Quantification Direct Kit (EpiGentek, Farmingdale, NY, USA). Briefly, DNA was extracted with the Dneasy Blood and Tissue Kit (Qiagen, Venlo, The Netherlands), and concentrations were determined using a Qubit 2.0 fluorometer (Invitrogen, Thermo Fisher Scientific). To measure 8-OhdG (8-hydroxy-2′-deoxyguanosine) levels, 300 ng of DNA was bound to a 96-well flat-bottom plate. Then, DNA samples were washed and incubated with the capture antibody (1:100). After washing, the detection antibody (1:1000) and the enhancer solution (1:5000) were added to DNA samples, followed by a color-developing solution to allow absorbance measurements at 450 nm using a VERSAmax^TM^ tunable microplate reader with SOFTmax^®^ Pro 3.0 software (Molecular Devices).

### 2.9. Protein Carbonylation

Levels of protein carbonylation were determined using the Oxyblot™ Kit (Millipore, Merck). In brief, one aliquot of protein extracts from each condition was derivatized with 2,4-dinitrophenylhydrazine (derivatization reaction), and a second aliquot was treated with a control solution (negative control). Carbonylated proteins were detected using a primary antibody specific for the dinitrophenylhydrazone residues, followed by an HRP-conjugated secondary antibody. Protein extraction, SDS-PAGE electrophoresis and immunodetection were performed as described above.

### 2.10. Transfection with mCherry-GFP-Tagged LC3 and Live-Cell Microscopy

Cells were seeded in glass-bottom plates and cultured as indicated. After 24 h, cells were transfected with the mCherry-GFP-LC3 reporter [50] using Lipofectamine 2000 (Thermo Fisher Scientific). The method is based on the inactivation of GFP fluorescence due to the acidification of lysosomes, whereas mCherry fluorescence remains unaffected, thus allowing the visualization of autophagosomes (puncta displaying green and red fluorescence) and autolysosomes (puncta displaying red fluorescence only). Progenitor HEI-OC1 cells were left either untreated or treated with IGF-1 or differentiation was triggered. Cellular fluorescence was imaged for up to 24 h using a Zeiss inverted Cell Observer microscope (Carl Zeiss). At the times indicated, cells were fixed, incubated with DAPI (1:1000, Thermo Fisher Scientific) and Alexa Fluor^®^ 647 Phalloidin (1:250; Thermo Fisher Scientific), mounted in ProLong^®^ Diamond (Thermo Fisher Scientific), and imaged using a Zeiss LSM710 confocal microscope (Carl Zeiss). The number of fluorescent bodies per cell in a total of 25 cells was quantified using Icy software [51]. The sum of all fluorescent particles is the total number of autophagic vesicles.

### 2.11. Statistical Analysis

Statistical significance was determined by one-way analysis of variance (ANOVA) or by two-tailed Student’s *t*-test after Levene’s or Fisher’s test of equality of variances, respectively, using SPSS v27.0 software (IBM, Armonk, NY, USA) or Microsoft Excel software (Microsoft, Redmond, WA, USA). Multiple comparison post hoc analyses included Bonferroni and Tamhane’s T2 tests when equal variances were assumed or not, respectively. Data are expressed as mean ± SEM. Results were considered significant at *p* < 0.05.

## 3. Results

### 3.1. HEI-OC1 Cells Display Characteristic Phenotypes of Progenitor and Differentiated Auditory Cells

**Figure 2:**
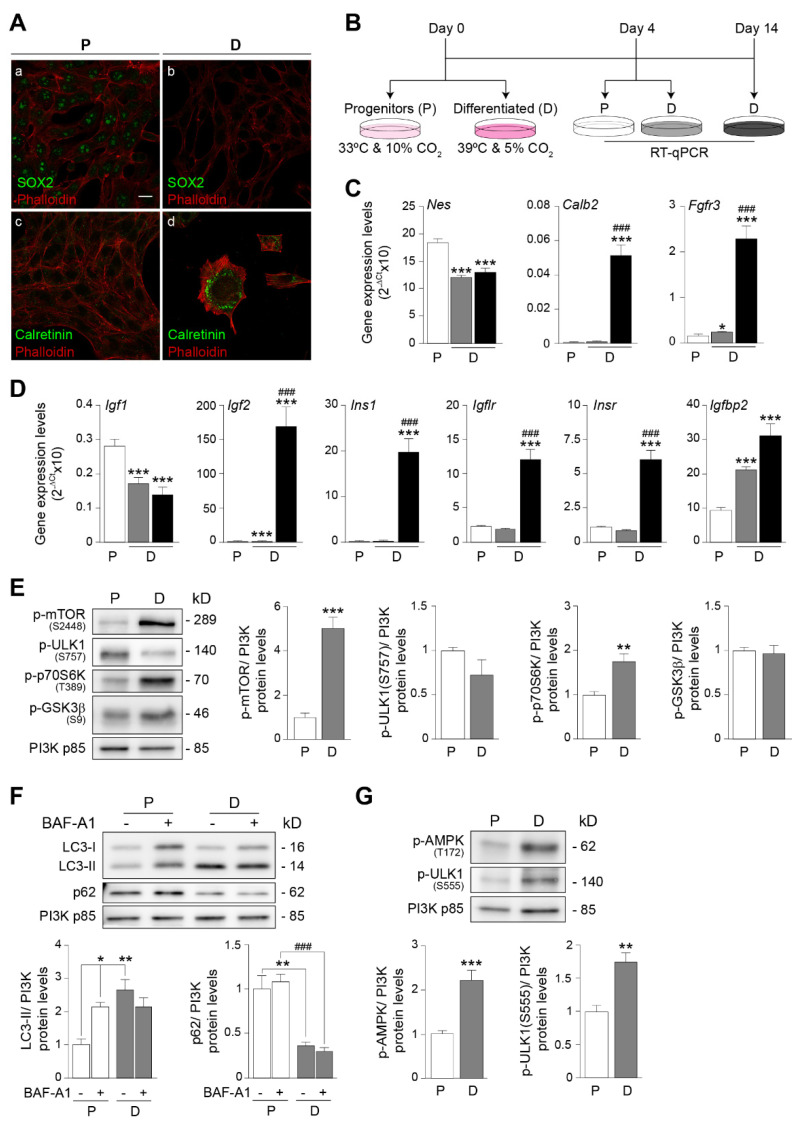
Progenitor and differentiated HEI−OC1 auditory cells show distinct metabolic profiles depending on the differentiation stage. (**A**) Representative confocal images of HEI−OC1 progenitor (P) and differentiated (**D**) auditory cells immunolabeled for SOX2 (**a**,**b**) and calretinin (**c**,**d**), both in green. Cells were incubated at 33 °C and 10% CO_2_ (HEI−OC1 P) or at 39 °C and 5% CO_2_ (HEI−OC1 D) for 4 days. Cellular cytoskeleton was stained with Alexa Fluor^®^ 546 phalloidin. Scale bar = 20 µm. (**B**) HEI−OC1 P cells were cultured for 4 days under proliferative conditions, and HEI−OC1 D cells were cultured for 4 or 14 days under differentiation conditions. Cells were then lysed, and the expression of target genes was analyzed by qPCR. (**C**,**D**) mRNA expression levels of *Nes*, *Calb2* and *Fgfr3* (**C**) and of *Igf1*, *Igf2*, *Ins1*, *Igf1r*, *Insr* and *Igfbp2* (**D**) were measured in HEI−OC1 P cultured for 4 days (white bars) and in HEI−OC1 D cells incubated for 4 (gray bars) or 14 (black bars) days. Gene expression levels were calculated as 2^−ΔCt^ using *Hprt1* as an endogenous housekeeping gene. Data are expressed as mean ± SEM from n = 3−11 independent samples measured in triplicate. Statistical significance was estimated by one-way ANOVA: * *p* < 0.05 and *** *p* < 0.001 versus HEI−OC1 P; ### *p* < 0.001 versus HEI−OC1 D for 4 days. (**E**) HEI−OC1 P (white bars) and HEI−OC1 D (gray bars) cells were cultured for 4 days. Western blotting was performed to assess p−mTOR (Ser2448), p−ULK1 (Ser757), p−p70S6K (Thr389) and p−GSK3β (Ser9) levels. (**F**) HEI−OC1 cells cultured as previously described were treated with BAF−A1 (100 nM) for 6 h and lysed for the immunodetection of LC3−II and p62. (**G**) HEI−OC1 cells cultured as previously described were lysed for the immunodetection of p−AMPK (Thr172) and p−ULK1 (Ser555). Representative Western blots from at least n = 4 independent samples per condition are shown. PI3K p85 was used as a loading control. Data are shown as mean ± SEM. Statistical significance was determined by Student’s t−test (** *p* < 0.01 and *** *p* < 0.001 versus HEI−OC1 P) or by one−way ANOVA (* *p* < 0.05 and ** *p* < 0.01 versus HEI−OC1 P; ### *p* < 0.001 versus HEI−OC1 P + BAF−A1), as indicated for two or more than two experimental groups, respectively.

HEI-OC1 cells were cultured under permissive (33 °C and 10% CO_2_) or non-permissive (39 °C and 5% CO_2_) conditions for 4 days and, accordingly, expressed, respectively, specific cellular markers of progenitor or differentiated auditory cells [43] (Figure 2, panels A–C). Progenitor cells displayed a stem-cell-like cell molecular phenotype characterized by the expression of the pluripotency marker SRY-Box transcription factor 2 (SOX) (Figure 2A(a,b)) and high levels of the neuroectodermal stem cell marker nestin (*Nes*) (Figure 2C), which declined with cell differentiation. In striking contrast, differentiated HEI-OC1 cells displayed a profile characterized by the expression of markers of mature organ of Corti cells, including calretinin (*Calb2*) (Figure 2C), encoding a calcium-binding protein whose expression is restricted to the perinuclear region (Figure 2A(c,d)), and fibroblast growth factor receptor 3 (*Fgfr3*) (Figure 2C), whose levels increased progressively with cell differentiation. These results confirmed the proliferative and post-mitotic cell phenotype of progenitor and differentiated HEI-OC1 cells, respectively, reported by Kalineck et al. [43,44].

The expression of IGF-system genes in the mouse inner ear is progressively restricted to specialized cell populations during differentiation [14,15]. We explored the expression pattern of IGF-system transcripts in proliferating and differentiated HEI-OC1 cells (Figure 2B,D), finding that *Igf1* expression levels decreased and *Igf2* and *Ins1* levels increased with cell differentiation. The expression of *Igf1r*, *Insr* and *Igfbp2* also increased with differentiation.

We next aimed to study the metabolic status of HEI-OC1 cells during their transition from proliferative to post-mitotic, differentiated cells by evaluating markers of active anabolic and catabolic pathways (Figure 2E–G). Progenitor cells were characterized by basal activation levels of mTOR signaling and autophagy, whereas differentiation triggered the activation of mTOR and its target p70S6K (5- and 1.8-fold increase, respectively) (Figure 2E). By contrast, no significant changes between proliferating and differentiated cells were observed in the levels of p-ULK1 (Ser757), an mTOR target that inhibits the induction of autophagy, or in the levels of p-GSK3β. 

Of note, molecular analysis revealed that anabolic (protein synthesis) pathways coexisted with catabolic (autophagy) pathways in differentiated HEI-OC1 cells. Specifically, the levels of the lipid-modified form of microtubule-associated protein 1 light chain 3 (LC3-II), which participates in autophagosome formation, were significantly higher in differentiated cells than in progenitors (2.6-fold), whereas the opposite was seen for the levels of p62 (also known as sequestome 1 or SQSTM1), which is degraded during autophagy [52] (Figure 2F). LC3-II levels did not increase after the addition of BAF-A1, an inhibitor of autophagosome-lysosome fusion [52,53], suggesting that LC3-II was elevated due to a block at the final stage of the autophagic flux. We next transfected progenitor HEI-OC1 cells with the autophagy flux reporter mCherry-GFP-LC3 [47] and imaged the cultures before and after the induction of differentiation. We found that the number of autophagic vesicles increased rapidly after the induction of differentiation (Supplementary Video S1). Quantification of confocal microscopy images at the end of the experiment revealed a higher number of autophagosomes in differentiated cells (1.8-fold) than in progenitors, although no differences were observed in the number of autolysosomes (Supplementary Figure S1A).

Finally, we studied the activation of AMPK and the phosphorylation of ULK1 on Ser555, which is targeted by AMPK to promote autophagy induction [54]. Results showed that ULK1 was activated in differentiated cells, presumably by p-AMPK, which phosphorylates Ser555 (Figure 2G), indicating that catabolic autophagy is upregulated in differentiated auditory cells.

### 3.2. IGF-1 Stimulation of IGF1R Activates Both AKT and ERK1/2 Signaling in Progenitor and Differentiated HEI-OC1 Auditory Cells

**Figure 3:**
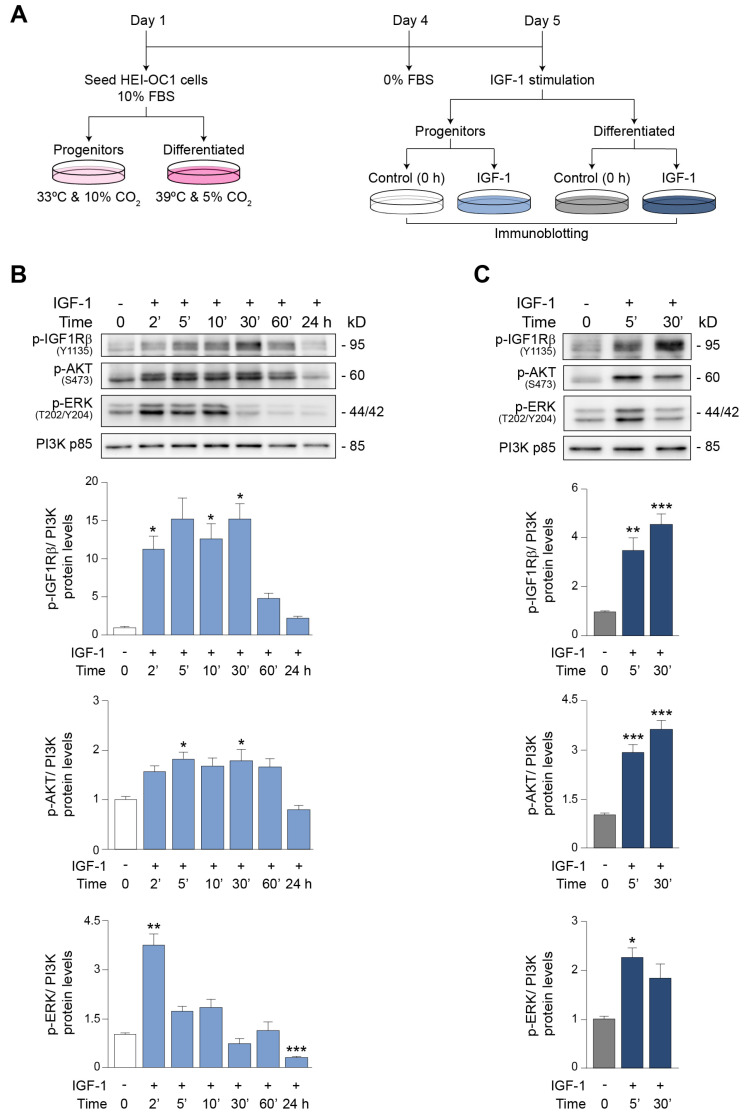
IGF−1 activates IGF1R and the downstream AKT survival and ERK proliferation pathways in a temporal manner. (**A**) HEI−OC1 P and D cells were cultured in DMEM with 10% FBS for 72 h, then switched to a control medium for 24 h and treated or not with IGF−1 (10 nM) for the times indicated, and lysed for the immunodetection of target proteins. (**B**,**C**) Levels of p−IGF1Rβ (Tyr1135), p−AKT (Ser473) and p−ERK (Thr202/Tyr204) measured by Western blotting in HEI−OC1 P cells either left untreated (white bars) or treated with IGF−1 (light blue bars) (**B**) and in HEI−OC1 D cells either left untreated (gray bars) or treated (dark blue bars) with IGF−1 (**C**) for the times indicated. Representative Western blots from n = 3 independent experiments performed in triplicate are shown. PI3K p85 was used as a loading control. Data are expressed as mean ± SEM. Statistical significance was determined by one−way ANOVA: * *p* < 0.05, ** *p* < 0.01 and *** *p* < 0.001 versus control (0 h).

IGF-1 downstream signaling is rapidly and transiently activated once IGF-1 interacts with IGF1R, triggering long-term effects in gene expression and cellular processes that are cell-type specific [33,55,56]. Thus, we next studied the downstream signaling pathways in progenitor and differentiated HEI-OC1 cells after IGF-1 treatment, following the experimental scheme described in Figure 3A and considering that HEI-OC1 cell differentiation triggers apoptosis, which limits the biological material available for further studies. Progenitor and differentiated cells were cultured in serum-free medium (control experimental group) for 24 h before treatment or not with IGF-1 to study temporal responses. The relative phosphorylation levels of the main downstream targets of IGF-1 (IGF1Rβ, AKT and ERK) were then examined by Western blotting in the protein extracts of progenitor (Figure 3B) and differentiated (Figure 3C) cells. 

Results showed a rapid and significant increase in the levels of p-IGF1Rβ (11-fold) in progenitor cells following IGF-1 treatment, which was sustained for 30 min. Similarly, levels of activated AKT increased 1.8-fold 5 min after IGF-1 exposure, which was maintained for up to 30 min. A fast but transitory activation of ERK (3.7-fold) was also observed 2 min following IGF-1 stimulation (Figure 3B). As shown in Figure 3C, the phosphorylation levels of IGF1Rβ, AKT and ERK also significantly increased in differentiated HEI-OC1 cells treated with IGF-1 for 5 min (3.5-, 2.9- and 2.3-fold, respectively), with a similar temporal profile to that of progenitors. Overall, the findings indicate that IGF-1 stimulation of HEI-OC1 cells results in the temporal activation of IGF1R and its main downstream targets.

### 3.3. IGF-1 Promotes Constructive Metabolism in Both Progenitor and Differentiated Auditory Cells but Differentially Modulates Autophagic Flux

**Figure 4:**
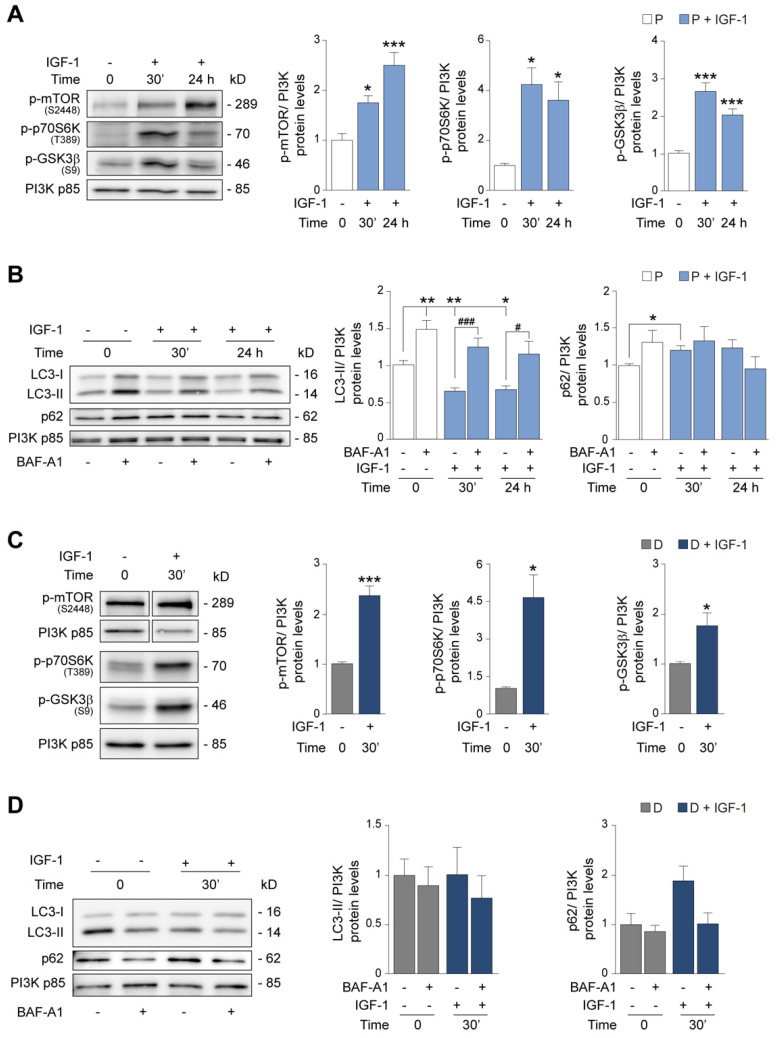
IGF−1 stimulates anabolism and reduces autophagic flux in HEI−OC1 progenitor cells**.** (**A**) HEI−OC1 P cells were cultured as shown in Figure 3A and left untreated (white bars) or treated (light blue bars) with IGF−1 (10 nM) for 30 min or 24 h and then lysed for the immunodetection of p−mTOR (Ser2448), p−p70S6K (Thr389) and p−GSK3β (Ser9). (**B**) HEI−OC1 P cells were starved for 24 h and left untreated (white bars) or treated (light blue bars) with IGF−1 (10 nM) for 30 min or 24 h, BAF−A1 (100 nM) for 6 h, or a combination of both, as indicated. Cells were lysed, and LC3−II and p62 levels were analyzed by Western blotting. Representative immunoblots from at least n = 3 independent experiments are shown. PI3K p85 was used as a loading control. Data are expressed as mean ± SEM. Statistical significance was determined using one−way ANOVA: * *p* < 0.05, ** *p* < 0.01 and *** *p* < 0.001 versus control (0 h); # *p* < 0.05 and ### *p* < 0.001 versus IGF−1 at the same experimental time. (**C**) HEI−OC1 D cells were cultured as shown in Figure 3A and left untreated (gray bars) or treated (dark blue bars) with IGF−1 (10 nM) for 30 min and lysed for the immunodetection of p−mTOR (Ser2448), p−p70S6K (Thr389) and p−GSK3β (Ser9). (**D**) HEI−OC1 D cells were starved for 24 h and left untreated (gray bars) or treated (dark blue bars) with IGF−1 (10 nM) for 30 min, BAF−A1 (100 nM) for 6 h, or a combination of both, as indicated. Cells were lysed, and LC3−II and p62 levels were analyzed by Western blotting. Representative immunoblots from at least n = 4 independent samples per condition are shown. PI3K p85 was used as a loading control. Immunoblots for p−mTOR (Ser2448) and its control came from non-consecutive lanes. Data are expressed as mean ± SEM. Statistical significance between the two experimental groups was determined by Student’s t−test: * *p* < 0.05 and *** *p* < 0.001 versus control (0 h).

We next studied the metabolic response to IGF-1 by Western blotting. Progenitor HEI-OC1 cells were treated with IGF-1 for 30 min or 24 h following the same experimental design shown in Figure 3A. Immunoblotting of cell extracts revealed a significant and progressive increase in p-mTOR (1.7-fold) levels following IGF-1 exposure. Likewise, the levels of p-p70S6K and p-GSK3β were significantly higher after 30 min of IGF-1 treatment and were decreased 24 h later (Figure 4A), indicating that IGF-1 upregulates anabolic pathways.

We next examined the modulatory role of IGF-1 on catabolic autophagy in progenitor HEI-OC1 cells stimulated with IGF-1 for 30 min or 24 h, with or without treatment with BAF-A1 for 6 h, by measuring the protein levels of autophagy markers (Figure 4B). As expected, BAF-A1 treatment led to an increase in LC3-II processing (1.5-fold) in the control (no IGF-1) experimental group. Contrastingly, LC3-II levels were significantly lower in IGF-1-treated cells (0.3-fold) than in control cells, but this did not occur in the presence of BAF-A1. A significant increase in p62 was also found after 30 min of IGF-1 exposure, although no relevant changes were observed in the presence of BAF-A1 (Figure 4B). As stated above, p62 is degraded by autophagy; thus, the total cellular levels of p62 are typically inversely correlated with LC3 and autophagosome formation [52,53]. To clarify the role of IGF-1 in the regulation of autophagic flux, we again used the mCherry-GFP-LC3 reporter [50], and transfected progenitor HEI-OC1 cells were cultured in the control medium and imaged before and after exposure to IGF-1 for 24 h. Results showed that the number of autophagosomes was lower in IGF-1-treated cells than in control cells, and autolysosomes were only visible at later incubation times (Supplementary Videos S2 and S3). Likewise, the number of autolysosomes was significantly lower in IGF-1-treated cultures (Supplementary Figure S1B), confirming the notion that IGF-1 causes both an inhibition of autophagosome production and a reduction in the autophagic flux.

IGF-1 treatment of differentiated HEI-OC1 cells for 30 min triggered the phosphorylation of mTOR (2.3-fold) and its main downstream target p70S6K (4.6-fold) and also triggered the phosphorylation of GSK3β (Figure 4C). No differences were observed in LC3-II or p62 levels in the presence of IGF-1 or BAF-A1 at the times studied. The limited number of differentiated HEI-OC1 cells did not allow for longer treatment times (Figure 4D). Taken together, the data suggest that IGF-1 promotes anabolism in differentiated HEI-OC1 auditory cells in contrast to the metabolic balance observed in progenitor cells, although the autophagic flux is maintained.

### 3.4. IGF-1 Stimulation of IGF1R Suppresses DNA Oxidative Damage and Apoptosis in HEI-OC1 Auditory Cells

**Figure 5:**
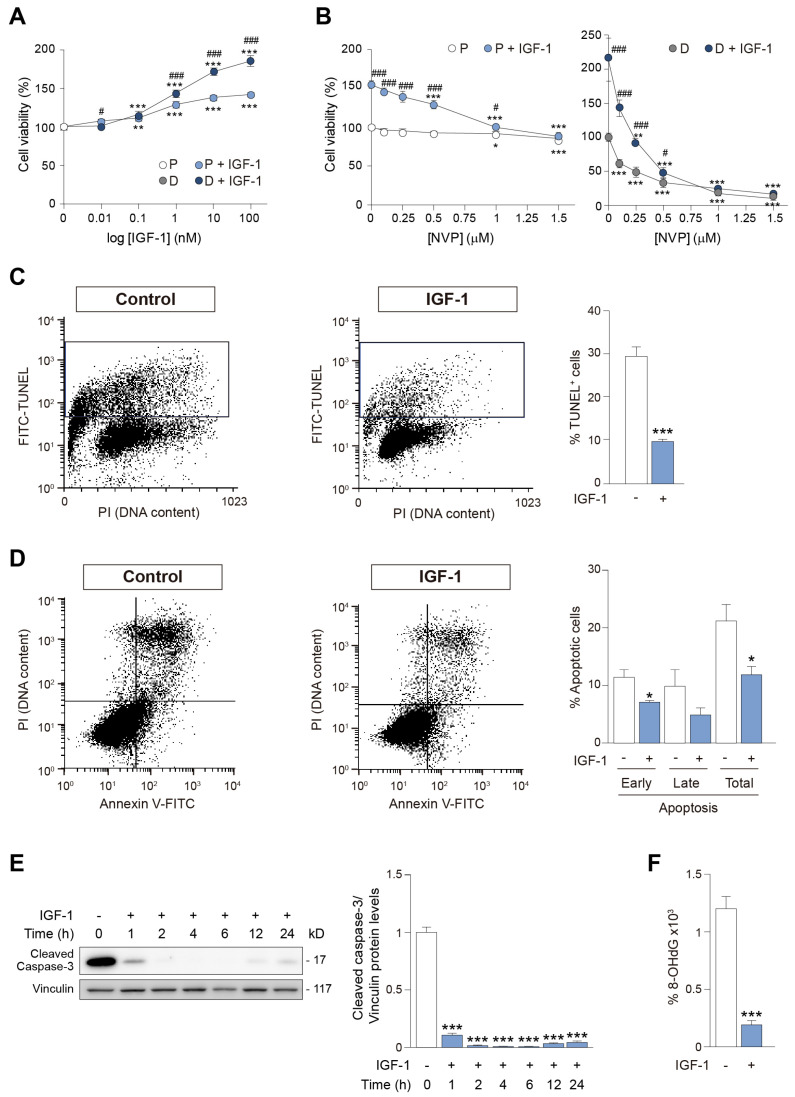
IGF−1 enhances cell survival through IGF1R and inhibits apoptosis and DNA oxidative damage in HEI−OC1 auditory cells. (**A**) HEI−OC1 P (white circles) and HEI−OC1 D (gray circles) cells were cultured in control medium for 24 h and then treated with different concentrations of IGF−1 (P, light blue circles; D, dark blue circles) for a further 24 h. Cell viability was measured by XTT assay. Average optical density measured in untreated control cells was taken as 100% of viability. Results are expressed as mean ± SEM from n = 2 independent experiments, with at least 5 independent samples per condition in each experiment. Statistical significance was estimated by Student’s t−test (### *p* < 0.001 versus HEI−OC1 P within same IGF−1 dose) or by one−way ANOVA (** *p* < 0.01 and *** *p* < 0.001 versus untreated control cells) when there were two or more than two experimental groups, respectively. (**B**) HEI−OC1 P (left panel) or HEI−OC1 D (right panel) cells were cultured as above, treated with the IGF1R inhibitor NVP at different concentrations for 2 h, and then treated (P, light blue circles; D, dark blue circles) or not (P, white circles; D, gray circles) with IGF−1 (10 nM) for 24 h. Cell viability was determined by XTT assay. Average optical density measured in untreated control cells was taken as 100% of viability. Results are expressed as mean ± SEM from n = 2 independent experiments, with at least 6 independent samples per condition in each experiment. Statistical significance was estimated by Student’s t−test (# *p* < 0.05 and ### *p* < 0.001 versus IGF−1-treated) or by one−way ANOVA (* *p* < 0.05, ** *p* < 0.01 and *** *p* < 0.001 versus NVP 0 µM) when there were two or more than two experimental groups, respectively. (**C**) HEI−OC1 P cells were cultured in control medium for 24 h and left untreated (white bars) or treated with IGF−1 (10 nM) (light blue bars) for a further 24 h. After treatment, TUNEL staining was used to detect DNA fragmentation, and TUNEL−positive cells were quantified by flow cytometry. Representative flow cytometry profiles from n = 5 independent samples are shown. (**D**) HEI−OC1 P cells treated as in (C) were stained with annexin V−FITC and propidium iodine (PI) to detect apoptotic cells by flow cytometry. Quantification of the early, late and total percentage of apoptosis in untreated (white bars) or IGF−1-treated (light blue bars) HEI−OC1 P cells is shown. Representative flow cytometry profiles from n = 2 independent experiments analyzed in triplicate are shown. Data are expressed as mean ± SEM. Statistical significance between the two experimental groups was determined by Student’s t−test: * *p* < 0.05 and *** *p* < 0.001 versus untreated control cells. (**E**) HEI−OC1 P cells were cultured in control medium for 24 h and left untreated (white bars) or treated with IGF−1 (10 nM) (light blue bars) for 1, 2, 4, 6, 12 or 24 h. Cells were then lysed for the immunodetection of cleaved caspase−3. Representative immunoblots from at least n = 4 independent samples are shown. Vinculin was used as a loading control. Data are expressed as mean ± SEM. Statistical significance was determined by one−way ANOVA: *** *p* < 0.001 versus untreated control cells. (**F**) HEI−OC1 P cells were cultured in control medium for 24 h and left untreated (white bars) or treated with IGF−1 (10 nM) (light blue bars) for a further period of 24 h. Cells were then lysed, DNA was extracted, and the percentage of 8−OHdG in the DNA was measured. Results are expressed as mean ± SEM from n = 3 independent samples per condition measured in triplicate. Statistical significance between the two experimental groups was estimated by Student’s t−test: *** *p* < 0.001 versus untreated control cells.

IGF-1 treatment enhanced the viability of both progenitor and differentiated HEI-OC1 cells in a dose-dependent manner (Figure 5A). The dose–response profile further suggested that IGF-1 acted through IGF1R [20]. To further confirm this observation, we treated cells with NVP, a selective inhibitor of IGF1R tyrosine kinase autophosphorylation and activation [57]. HEI-OC1 cells were pre-treated with increasing concentrations of NVP for 2 h before treatment with IGF-1 for 24 h (Figure 5B). Results showed that NVP alone had no influence on the viability of HEI-OC1 progenitor cells, but it dose-dependently suppressed the protective effect of IGF-1 (Figure 5B, left panel). Contrastingly, cell viability was lower in NVP-pre-treated differentiated HEI-OC1 cells both in the absence and presence of IGF-1 (Figure 5B, right panel), suggesting that differentiated auditory cells are highly dependent on IGF-1 levels.

To further study the protective effects of IGF-1 on HEI-OC1 progenitors, cells were treated with IGF-1 for 24 h, labeled by TUNEL and analyzed by flow cytometry. Results showed that IGF-1 significantly reduced (0.66-fold) the percentage of TUNEL-positive cells (Figure 5C). We confirmed this using annexin V-FITC/PI double staining, which revealed a decrease in early apoptotic cells and an overall decrease in total apoptosis in HEI-OC1 progenitor cells following IGF-1 treatment (Figure 5D). Aligning with this, IGF-1 significantly decreased (0.9-fold) the levels of cleaved caspase-3 (Figure 5E). 

IGF-1 has been reported to serve an antioxidant role in multiple cellular contexts, such as vascular tissue [58]. We thus measured the levels of the major oxidant product 8-OHdG in DNA in progenitor cells treated or not with IGF-1 for 24 h. Results showed that the percentage of 8-OHdG in DNA was significantly lower in IGF-1-treated cells (0.84-fold) than in control cells (Figure 5F). Overall, these results support the notion that IGF-1 has antioxidant and antiapoptotic properties in HEI-OC1 auditory cells.

### 3.5. IGF-1 Partially Protects against the Early- and Long-Term Ototoxic Effects of Cisplatin in HEI-OC1 Progenitor Cells

To further explore the homeostatic functions of IGF-1 and its potential as an otoprotector, we used cisplatin as an ototoxic insult because it is known to induce oxidative stress in hair cells [38]. Cisplatin enters cells by passive diffusion and active transport and has both short- and long-term differential actions, with the latter including DNA damage, induction of oxidative stress and apoptosis [46]. Because HEI-OC1 differentiation triggers apoptosis, which limits the experimental design, the study of cytotoxic drugs has been typically limited to progenitors [43,44]. As cisplatin is toxic for post-mitotic hair cells in vivo, we used serum deprivation as an experimental model to study cisplatin under non-proliferative conditions in progenitor cells.

**Figure 6:**
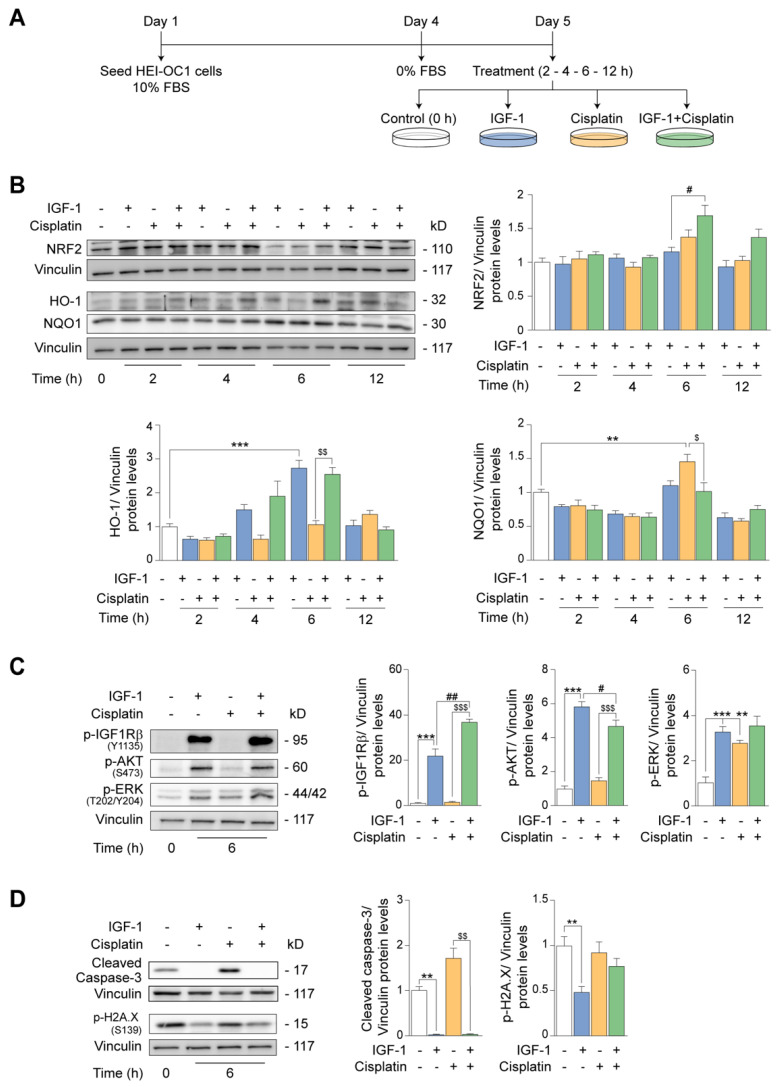
Ototoxic effects induced by short−term exposure to cisplatin are neutralized by IGF−1. (**A**) HEI−OC1 P cells were cultured in DMEM with 10% FBS for 72 h and then switched to a control medium for 24 h and left untreated (white bars) or treated with IGF−1 (10 nM) (light blue bars), cisplatin (4 µg/mL) (yellow bars) or a combination of both (green bars) for 2, 4, 6 and 12 h and lysed for the analysis of target proteins. (**B**) Levels of NRF2, HO−1 and NQO1 were measured by Western blotting. Representative immunoblots from at least n = 4 independent experiments are shown. (**C**) Levels of p−IGF1Rβ (Tyr1135), p−AKT (Ser473) and p−ERK (Thr202/Tyr204) were analyzed by Western blotting. Representative immunoblots from at least n = 4 independent experiments are shown. (**D**) Levels of cleaved caspase−3 and p−H2A.X (Ser139) were measured by Western blotting. Representative immunoblots from at least n = 3 independent experiments are shown. Vinculin was used as a loading control. Data are expressed as mean ± SEM. Statistical significance was determined by one−way ANOVA: ** *p* < 0.01 and *** *p* < 0.001 versus control (0 h); # *p* < 0.05 and ## *p* < 0.01 versus IGF−1; $ *p* < 0.05, $$ *p* < 0.01 and $$$ *p* < 0.001 versus cisplatin.

Cells were cultured for 24 h and were treated or not with IGF-1, cisplatin or a combination of both for 2–12 h to study early intracellular signaling responses (Figure 6A). We then used Western blotting to assess the levels of nuclear factor (erythroid-derived 2)-like 2 (NRF2), a master regulator of anti-oxidative responses, and its main targets, heme oxygenase 1 (HO-1) and NAD(P)H:quinone oxidoreductase 1 (NQO1) [59]. Under these conditions, IGF-1 and cisplatin co-treatment for 6 h induced a significant increase (1.5-fold) in the levels of NRF2 over that observed with IGF-1 alone and a 2.4-fold increase in the levels of HO-1. Additionally, co-treatment with IGF-1 reduced (0.4-fold) the increase in NQO1 levels stimulated by cisplatin alone (Figure 6B). Based on these findings, we selected the 6-h time-point for further analysis of early cisplatin effects on auditory cells.

In the presence of cisplatin for 6 h, IGF-1 activated IGF1R-mediated AKT and ERK signaling pathways. Cisplatin treatment alone activated ERK to the same extent as IGF-1, and no changes were observed by co-treatment after 6 h of incubation. Of note, IGF1R activation was increased (1.7-fold) by IGF-1 and cisplatin co-treatment, whereas AKT activation was slightly reduced (0.2-fold) with respect to IGF-1 treatment alone (Figure 6C). IGF-1 treatment significantly decreased (0.9-fold) the levels of activated caspase-3, both alone and in combination with cisplatin (Figure 6D). Additionally, IGF-1 markedly reduced the levels of phosphorylated histone 2A.X (p-H2A.X) (0.5-fold), a DNA damage marker, but no evident changes were observed when cells were exposed to cisplatin alone or with IGF-1 for 6 h (Figure 6D).

**Figure 7:**
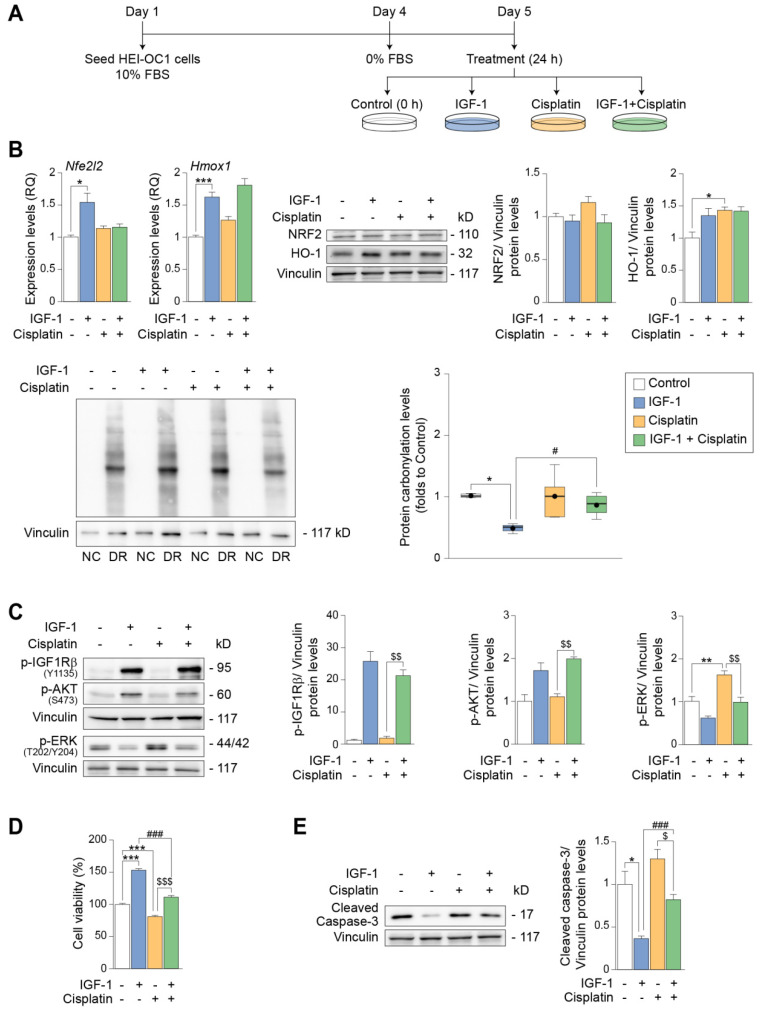
IGF−1 protects from ototoxic damage induced by long−term exposure to cisplatin. (**A**) HEI−OC1 P cells were cultured in DMEM with 10% FBS for 72 h and then switched to a control medium for 24 h and left untreated (white bars) or treated with IGF−1 (10 nM) (light blue bars), cisplatin (4 µg/mL) (yellow bars) or a combination of both (green bars) for 24 h and then lysed for the analysis of target genes and proteins. (**B**) mRNA expression levels of *Nfe2l2* and *Hmox1* were measured by qPCR (left upper panel), and protein levels of NRF2 and HO−1 were determined by Western blotting (right upper panel). Gene expression levels from n = 3 independent samples analyzed in triplicate were calculated as 2^−ΔΔCt^ using *Rplp0* as an endogenous gene. Representative immunoblots from at least n = 4 independent experiments, using vinculin as a loading control, are shown. Data are expressed as mean ± SEM. Oxidative protein carbonylation levels were measured with the Oxyblot^TM^ Kit (lower panel). Representative immunoblot with carbonylated proteins from n = 4 independent experiments, using vinculin as a loading control, is shown. Non−derivatized extracts (NC) are shown together with derivatized protein extracts (DR). Data are presented as a box plot, the mean value is plotted as a filled black circle and whiskers represent min and max values. Statistical significance was determined by one−way ANOVA: * *p* < 0.05 and *** *p* < 0.001 versus control (0 h); # *p* < 0.05 versus IGF−1. (**C**) Levels of p−IGF1Rβ (Tyr1135), p−AKT (Ser473) and p−ERK (Thr202/Tyr204) were measured by Western blotting. Representative immunoblots from at least n = 3 independent experiments are shown. Vinculin was used as a loading control. Data are expressed as mean ± SEM. Statistical significance was determined by one−way ANOVA: ** *p* < 0.01 versus control (0 h); $$ p < 0.01 versus cisplatin. (**D**) HEI−OC1 P cells were cultured in a control medium for 24 h and then treated with IGF−1 (10 nM), cisplatin (4 µg/mL) or a combination of both for a further period of 24 h. Cell viability was measured with the XTT assay. Average optical density measured in untreated control cells was taken as 100% of viability. Results are expressed as mean ± SEM from n = 4 independent experiments, and each experiment contained at least 4 independent samples per condition. Data are expressed as mean ± SEM. Statistical significance was determined by one−way ANOVA: *** *p* < 0.001 versus control (0 h); ### *p* < 0.001 versus IGF−1; $$$ *p* < 0.001 versus cisplatin. (**E**) Levels of cleaved caspase−3 were determined by Western blotting. Representative immunoblots from n = 4 independent experiments, using vinculin as a loading control, are shown. Data are expressed as mean ± SEM. Statistical significance was determined by one−way ANOVA: * *p* < 0.05 versus control (0 h); ### *p* < 0.001 versus IGF−1; $ *p* < 0.05 versus cisplatin.

Cytotoxic effects of cisplatin are observed at longer treatment times; therefore, we studied the response of HEI-OC1 cells exposed to IGF-1 or cisplatin, alone or in combination, for 24 h (Figure 7A). Under these conditions, IGF-1 treatment alone significantly increased (1.5-fold) the levels of *Nfe2l2* and *Hmox1*, coding, respectively, for NRF2 and HO-1, although this was not reflected at the protein level (Figure 7B). IGF-1 also lowered (0.5-fold) the oxidative protein carbonylation levels of HEI-OC1 cells (Figure 7B). Cisplatin treatment alone increased the protein levels of HO-1 (1.4-fold), but no other significant changes were observed in the other conditions tested. IGF-1 co-treatment was unable to influence protein carbonylation induced by 24 h exposure to cisplatin (Figure 7B). IGF-1 activated IGF1R and AKT in the presence of cisplatin (Figure 7C), and cell viability increased when cisplatin and IGF-1 were combined with respect to cisplatin alone, which decreased cell viability (Figure 7D). Notably, the cisplatin-sustained activation of ERK (1.6-fold) was suppressed by co-treatment with IGF-1 (Figure 7C). It has been reported that ERK activation has different outcomes depending on whether it is transitory or sustained. Indeed, the sustained activation of ERK is a major mediator of cisplatin-induced ototoxicity and leads to cell death [60,61]. Finally, IGF-1 treatment significantly decreased the levels of activated caspase-3, both alone (0.6-fold) and in combination with cisplatin (0.4-fold). Nevertheless, IGF-1-mediated protection was partial and did not completely prevent the activation of caspase-3 induced by 24 h exposure to cisplatin (Figure 7E). These results, taken together with the IGF-1-induced increase in cell viability, indicate that IGF-1 counteracts the ototoxic effects of cisplatin in HEI-OC1 cells.

## 4. Discussion

IGF-1 is a well-known modulator of brain metabolic homeostasis [1]. It has also been reported to be a key neurotrophic factor for the developing inner ear [27,28,29] during postnatal cochlear differentiation [8,9,14], with aging [10,62], and also in response to insults [4,12]. The underlying molecular mechanisms for this plethora of actions are not fully understood, and it is not known whether the capacity of IGF-1 to modulate metabolism plays a role in this context.

HEI-OC1 progenitor cells are characterized by a proliferative cell state, with the high expression of the pluripotency transcription factor Sox2 [63] and the neuroepithelial stem cell marker nestin [64]. By contrast, differentiated HEI-OC1 cells have a profile of mature organ of Corti cells, characterized by the expression of *Calb2* [65] and *Fgfr3* [66,67]. The high expression levels of calretinin (*Calb2*) are a trait of post-mitotic hair cells [68,69]. We found that IGF-system genes were differentially expressed in both progenitor and differentiated cells, confirming their tight regulation in the mammalian organ of Corti [12,14,15,16,17,18,19]. IGF-1 treatment of HEI-OC1 cells led to the rapid phosphorylation of IGF1R, AKT and ERK in both progenitor and differentiated populations but with distinct temporal profiles. The effects of IGF-1 on HEI-OC1 cells were mediated by its binding to IGF1R and the activation of pro-survival AKT signaling. We found that IGF1R signaling attenuated DNA oxidative damage, augmented cell viability and blunted apoptosis. The increase in cell survival in differentiated HEI-OC1 cells was striking, suggesting that IGF-1 is crucial for the maintenance of mature hair cells.

A comparative analysis of the metabolic status of both cellular stages revealed that IGF-1 activation of AKT and mTORC1 in progenitor cells increases biosynthetic routes, as reported in other cellular contexts [34], while reducing autophagic flux. Although the degradation of p62 is considered a typical read-out of autophagy, we did not generally observe significant changes in p62 levels. Because p62 can also be degraded by the proteasome, is subject to transcriptional regulation and is involved in other cellular processes, it is not always an informative marker of autophagy [53].

Differentiated HEI-OC1 cells also showed activated mTOR, p70S6K and GSK3β, which aligns with the reported anabolic state of differentiated cells and the required remodeling of the protein profile [66,67]. Concurrently, however, differentiated cells need to activate catabolic pathways, such as autophagy, to recycle amino acids and other intermediates to fuel the biosynthesis of new macromolecules prior to cell remodeling as well as to eliminate unwanted proteins [70]. Although autophagy is highly stimulated in differentiated HEI-OC1 cells, we failed to find higher levels of LC3-II after treatment with BAF-A1, suggesting that the increase in LC3-II levels could be due to either a blockade in a late step of the autophagic flux or to difficulties in resolving the increased autophagic flux [52,53]. Thus, we studied the activation of AMPK, a main inductor of autophagy [71], finding that AMPK and its target p-ULK1 (S555) were activated, thus stimulating catabolic autophagy. Both the activation of AMPK/ULK1 and the presence of autolysosomes in differentiated HEI-OC1 cells indicated that autophagy is massively induced during auditory cell differentiation. These data are consistent with previous reports showing that autophagy is upregulated during cell renewal processes [72,73]. Autophagy is crucial in the postnatal cochlea [74,75,76,77], which suggests that the induction of autophagy triggered by hair cell differentiation is resolved when differentiation is complete.

IGF-1 is otoprotective against cochlear insults, such as aminoglycosides [22,23,41,42], noise exposure [12,78] or ischemia [79]. Moreover, IGF-1 is crucial for the maintenance of cochlear synapses [80,81], and it has been approved for the treatment of human sudden sensorineural hearing loss refractory to corticosteroid treatment [82]. To the best of our knowledge, no studies have explored the potential otoprotective role of IGF-1 against cisplatin-related ototoxicity. Interestingly, in an albino rat model of cisplatin-induced nephrotoxicity, treatment with human growth hormone, which physiologically induces IGF-1 secretion, was protective by increasing IGF-1 expression and stimulating the antioxidant responses mediated by NRF2 and HO-1 [83]. We show here that IGF-1 likely counteracts the toxic effects of cisplatin in HEI-OC1 cells by inducing HO-1, a main target of NRF2, and suppressing protein oxidation, as reported in other cellular contexts [84,85]. We also found that cisplatin increased the levels of NQO1 (a target of NRF2), likely triggered as an intrinsic cellular antioxidant defense mechanism, as NQO1 is crucial for hearing protection against cisplatin [86]. 

IGF-1 induces the rapid and transitory activation of downstream signaling. By contrast, cisplatin has both short- and long-term actions. We found that cisplatin induced ERK activation for up to 24 h. Transitory ERK activation in the cochlea is an otoprotective mechanism [87,88,89], although its sustained activation promotes hair cell death [90]. Sustained ERK activation forms part of the mechanisms triggered by cisplatin to induce apoptosis [60,61,91]. IGF-1 could counteract cisplatin-mediated ERK activation, likely contributing to the protection of HEI-OC1 cells. We also found that IGF-1 induced IGF1R and AKT activation in the presence of cisplatin. AKT activation is a critical step in the cellular response to apoptosis, and we found that IGF-1 sustains AKT activation in HEI-OC1 cells exposed or not to cisplatin. Our data are consistent with previous reports demonstrating the pro-survival and otoprotective role of AKT signaling against cisplatin ototoxicity [85,86].

Finally, IGF-1 treatment also reduced the cleavage of caspase-3 and the levels of p-H2A.X, which are indices of apoptosis [92] and DNA damage [93], respectively, after long-term exposure to cisplatin. Cleaved caspase-3 is a read-out of apoptosis. We used serum deprivation as a model for non-proliferative conditions, which causes apoptosis in HEI-OC1 cells. Accordingly, the experimental design took into account the cisplatin-independent basal level of apoptosis at the times studied and also cisplatin-induced apoptosis at longer times. IGF-1 reduced serum-deprivation-induced apoptosis and partially prevented cisplatin-induced apoptosis.

## 5. Conclusions

We provide new insight into the role of IGF-1 as a central regulator of metabolic and oxidative homeostasis in sensory hair cells. We demonstrate here, for the first time, that IGF-1 is modulating energy balance by driving anabolism and reducing autophagic flux through IGF1R engagement in progenitor auditory cells. Remarkably, differentiated auditory cells require the upregulation of both mTORC1-mediated anabolic pathways and autophagy driven by AMPK/ULK1 axis to support cell turnover rate prior to complete differentiation. Interestingly, IGF-1 is not able to suppress the induction of autophagy observed when auditory differentiation is triggered. In a model of ototoxicity induced by cisplatin, IGF-1 protects auditory cells from oxidative stress and apoptosis through the IGF1R/AKT axis supporting the antioxidant cellular response. Finally, our data improve knowledge on the molecular and cellular mechanisms involved in hearing loss due to IGF-1 deficiency.

## Data Availability

Publicly available datasets were analyzed in this study. This data can be found on the following website: [http://hdl.handle.net/10261/279459] (Accessed on 20 September 2022).

## Supplementary Materials

The following supporting information is available online at https://www.mdpi.com/article/10.3390/antiox12020233/s1. Figure S1: IGF-1 interrupts autophagic flux in HEI-OC1 progenitors but not in differentiated cells; Video S1: Autophagic flux is increased when auditory cell differentiation is triggered; Video S2: Autophagic vesicle formation is induced in HEI-OC1 progenitors after serum withdrawal; Video S3: IGF-1 treatment inhibits the formation of new autophagic vesicles in HEI-OC1 progenitors; Table S1: List of antibodies used in immunofluorescence (IF) and Western blotting (WB) experiments; Table S2: List of TaqMan® probes used in RT-qPCR experiments; Table S3: List of specific primers designed for RT-qPCR experiments with SYBR Green.
