# Supplementary material for: IGF-1 Controls Metabolic Homeostasis and Survival in HEI-OC1 Auditory Cells through AKT and mTOR Signaling
Source: Antioxidants (Basel). 2023 Jan 19;12(2):233. doi: 10.3390/antiox12020233 (PMC9952701; doi:10.3390/antiox12020233)
Supplement: Supplementary file 1 [file antioxidants-12-00233-s001.zip › antioxidants-2156550 Supplementary Materials.pdf]

antioxidants

MDPI

Supplementary Materials

Scheme 1. [Movie]. Autophagic flux is increased when auditory cell differentiation is triggered. HEI-OC1 P cells were seeded in glass-bottom plates and cultured for 24 h. Cells were then transfected with mCherry-GFP-LC3 in control medium, and imaged for 1 h before the culture conditions were modified to allow progenitors to differentiate; cells were then imaged for a further 24 h. A representative video of mCherry-GFP-LC3-transfected HEI-OC1 cells from n=12 independent samples per condition is shown. Scale bar=20 µm.

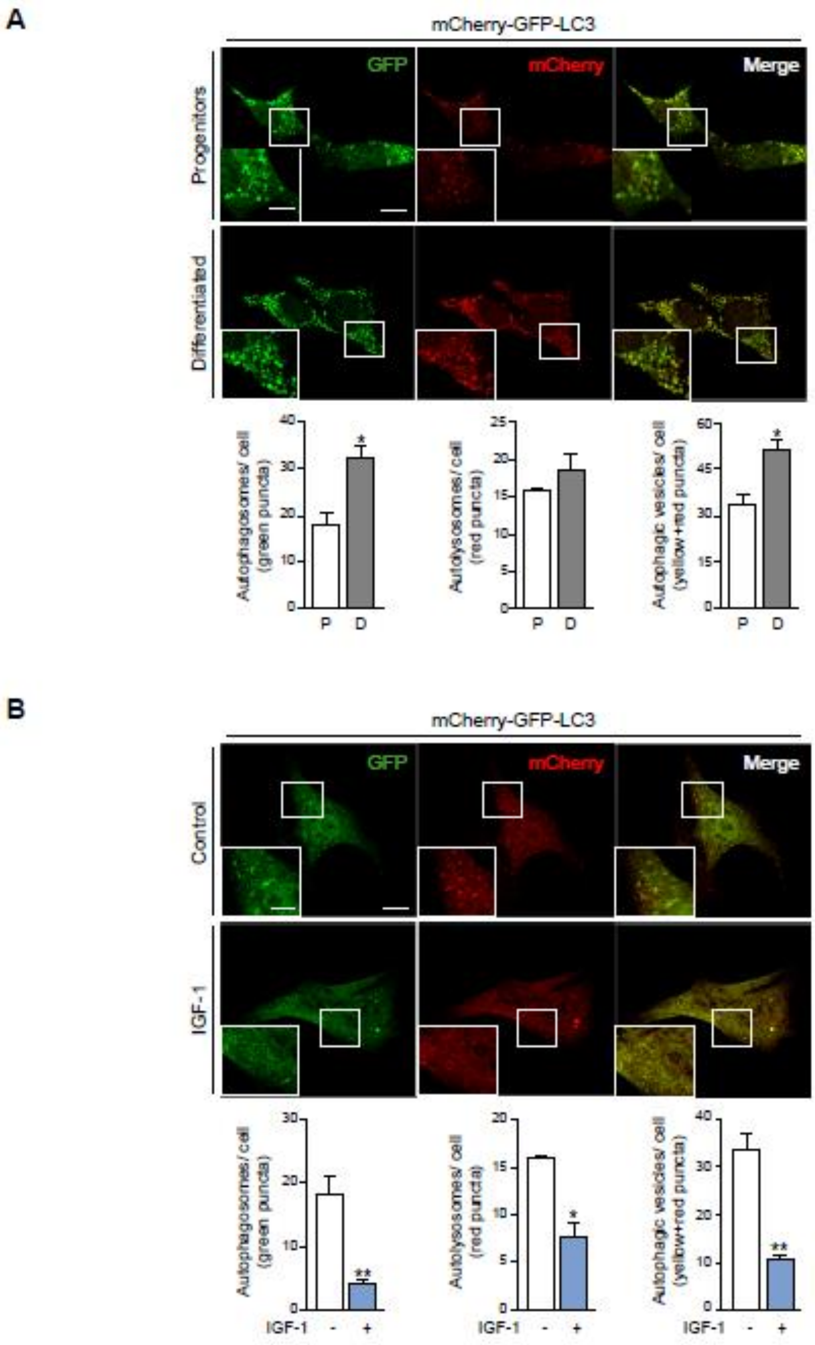

Figure S2. IGF-1 interrupts autophagic flux in HEI-OC1 progenitors but not in differentiated cells. A) HEI-OC1 P cells were seeded in glass-bottom plates for 24 h. Cells were then transfected with mCherry-GFP-LC3 and imaged for 24 h at 33ºC and 10% CO_2 (P), or at 39ºC and 5% CO_2 (D). At the end of the experiment, cells were fixed and stained. Representative confocal images of mCherry-GFP-LC3-transfected HEI-OC1 P and D cells from n=3 independent samples per condition

is shown (upper panel). **B)** HEI-OC1 P cells were cultured in glass-bottom plates for 24 h. Cells were transfected with mCherry-GFP-LC3 and treated or not with IGF-1 (10 nM) and imaged for a further 24 h. Cells were then fixed and stained. Representative confocal images of mCherry-GFP-LC3-transfected HEI-OC1 P cells from n=3 independent samples per condition are shown (upper panel). White squares show the enlarged area. Scale bars=10 μm and 5 μm (insets), respectively. Quantification of autophagosomes, autolysosomes and autophagic vesicles per cell, from a total of n=25 cells counted for each condition is shown (lower panel). Data are represented as mean±SEM of the number of fluorescent puncta per cell. Statistical significance between the two experimental groups was determined by Student's t-test: *p<0.05 *versus* HEIOC1 P (**A**) or *p<0.05 *versus* untreated control cells (**B**).

Scheme 3. [Movie]. Autophagic vesicle formation in HEI-OC1 progenitors is induced after serum withdrawal. HEI-OC1 P cells were seeded in glass-bottom plates for 24 h and transfected with mCherry-GFP-LC3. Cells were then imaged for 24 h. Representative videos of mCherry-GFP-LC3-transfected HEI-OC1 P cells from n=6 independent samples per condition are shown. Scale bars=10 μm.

Scheme 4. [Movie]. IGF-1 treatment inhibits the formation of new autophagic vesicles in HEI-OC1 progenitors. HEI-OC1 P cells were seeded in glass-bottom plates for 24 h and transfected with mCherry-GFP-LC3. Cells were then treated with IGF-1 (10 nM) and imaged for 24 h. Representative videos of mCherry-GFP-LC3-transfected HEI-OC1 P cells from n=6 independent samples per condition are shown. Scale bars=10 μm.

Table S1. List of antibodies used in immunofluorescence (IF) and western blotting (WB) experiments.

Target	Dilution	Technique	Manufacturer	Catalog Nº
**Calretinin**	1:100	IF	Abcam	#ab133316
**Cleaved Caspase-3**	1:1000	WB	Cell Signaling Technology	#9661
**HO-1**	1:1000	WB	Millipore	#AB1284
**LC3A/B**	1:1000	WB	Cell Signaling Technology	#4108
**NQO1**	1:1000	WB	Abcam	#ab2346
**NRF2**	1:1000	WB	Homemade	From A Cuadrado's lab (IIB "Alberto Sols", Madrid, Spain)
**p62**	1:1000	WB	MBL	#PM045
**p-AKT**	1:1000	WB	Cell Signaling Technology	#9271
**p-AMPKα**	1:1000	WB	Cell Signaling Technology	#2531
**p-ERK1/2**	1:1000	WB	Cell Signaling Technology	#9101
**p-GSK3β**	1:1000	WB	Cell Signaling Technology	#5558
**p-Histone H2A.X**	1:1000	WB	Cell Signaling Technology	#2577
**PI3K p85**	**1:5000**	**WB**	**Homemade**	**From AM Valverde's lab (IIB "Alberto Sols", Madrid, Spain)**
**p-IGF-1 Receptor β**	1:1000	WB	Cell Signaling Technology	#3918

**p-mTOR**	1:1000	WB	Cell Signaling Technology	#2971
**p-p70 S6 Kinase**	1:1000	WB	Cell Signaling Technology	#9234
**p-ULK1 (Ser 555)**	1:1000	WB	Cell Signaling	#5869
**p-ULK1 (Ser 757)**	1:1000	WB	Cell Signaling Technology	#14202
**SOX2**	1:100	IF	Abcam	#ab97959
**Vinculin**	1:15000	WB	Santa Cruz	#sc-73614
**Alexa Fluor® 647 Phalloidin**	1:250	IF	Thermo Fisher Scientific	#A22287
**Alexa Fluor® 546 Phalloidin**	1:250	IF	Thermo Fisher Scientific	#A22283
**Goat IgG**	1:5000	WB	Bio-Rad Laboratories	#1721034
**Mouse IgG**	1:3000	WB	Bio-Rad Laboratories	#1706516
**Rabbit IgG**	1:3000	WB	Bio-Rad Laboratories	#1706515
**Rabbit IgG Alexa Fluor® 488**	1:500	IF	Thermo Fisher Scientific	#A-11034

Table S2. List of TaqMan® probes used in RT-qPCR experiments.

**Symbol**	**Gene Name**	**Catalog Nº**
Igf1	Insulin-like growth factor 1	Mm00439561_m1
Igf2	Insulin-like growth factor 2	Mm00439564_m1
Igf1r	Insulin-like growth factor receptor 1	Mm00802831_m1
Igfbp2	Insulin-like growth factor binding protein 2	Mm00492632_m1
Ins1	Insulin 1	Mm01950294_s1
Insr	Insulin receptor (IR-A and IR-B)	Mm01211875_m1

Table S3. List of gene-specific primers designed for RT-qPCR experiments with SYBR Green.

**Symbol**	**Gene Name**	**Forward primer (5′-3′)**	**Reverse primer (5′-3′)**
Calb2	Calbindin 2	GCCGACCGAAGAGAATTTCC	CGCCAAGCCTCCATAAACTC
Fgfr3	Fibroblast growth factor receptor 3	CGACAGGTGTCCTTGGAATCTAA	CGGGCAATCCGGACAA
Hmox1	Heme oxygenase 1	GAGGCTAAGACCGCCTTCCT	TTGTGTTCCTCTGTCAGCATCAC
Nes	Nestin	TTCCTGACCCCAAGCTGAAG	TCACAGGAGTCTCAAGGGTATTAGG
***Nfe2l2* (NRF2)**	Nuclear factor erythroid 2 like 2	GCCTTGTACTTTGAAGACTGTATGCA	AAGCGACTCATGGTCATCTACAAAT
