# Supplementary figures and images for: IGF-1 Controls Metabolic Homeostasis and Survival in HEI-OC1 Auditory Cells through AKT and mTOR Signaling
Source: Antioxidants (Basel). 2023 Jan 19;12(2):233. doi: 10.3390/antiox12020233 (PMC9952701; doi:10.3390/antiox12020233)

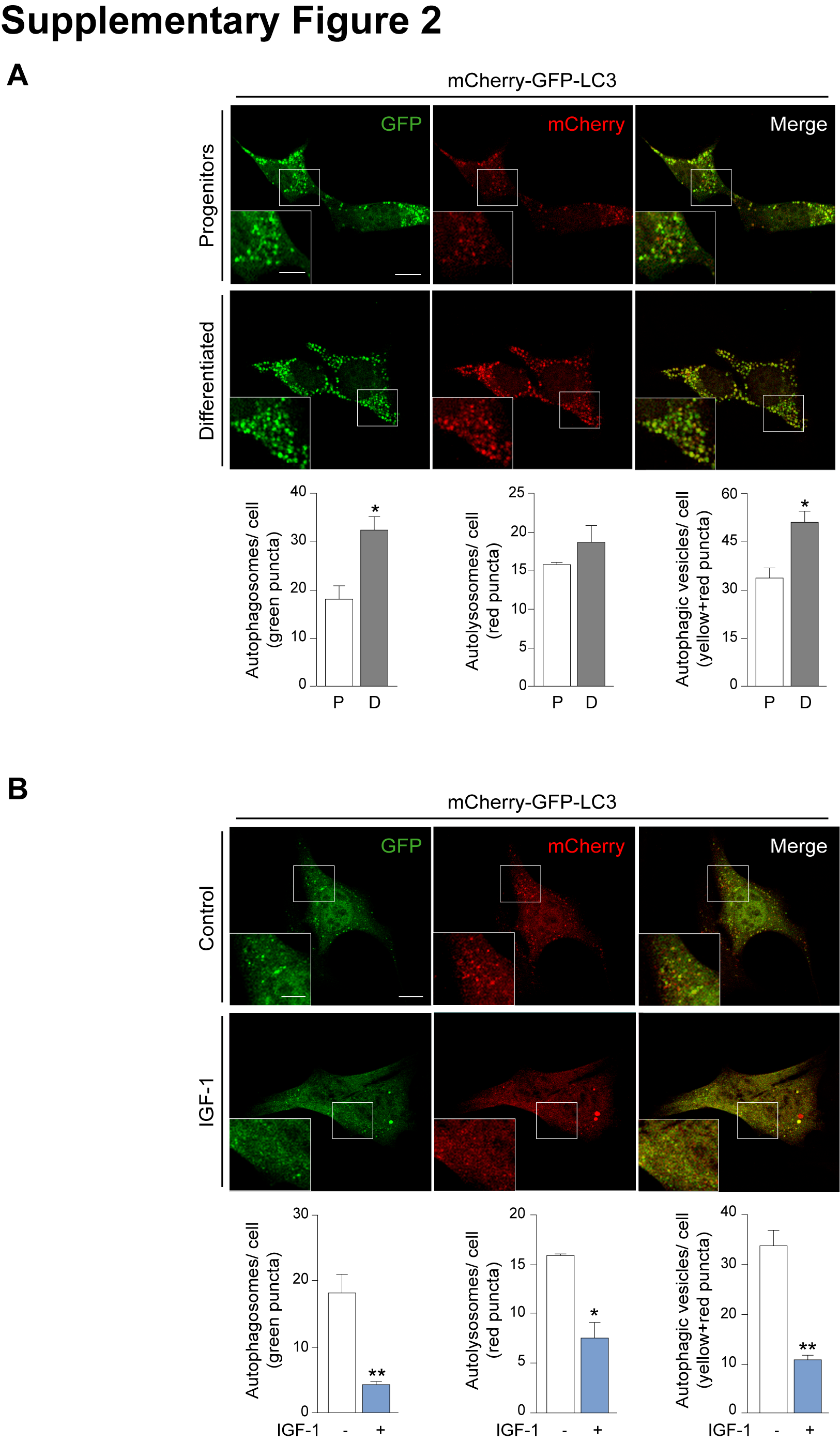

Supplement: Supplementary file 1 [file antioxidants-12-00233-s001.zip › Garcia-Mato et al_2022_Supplementary Figure S2_Antioxidants.tif]
